# Bioprospecting of *Artemisia* genus: from artemisinin to other potentially bioactive compounds

**DOI:** 10.1038/s41598-024-55128-z

**Published:** 2024-02-27

**Authors:** Stefano Negri, Fabio Pietrolucci, Sebastiano Andreatta, Ruth Chinyere Njoku, Carolina Antunes Silva Nogueira Ramos, Massimo Crimi, Mauro Commisso, Flavia Guzzo, Linda Avesani

**Affiliations:** 1 Department of Biotechnology, University of Verona, 15, Strada Le Grazie, 37134 Verona, Italy; 2 National Biodiversity Future Center (NBFC), 90133 Palermo, Italy; 3 Museo di Storia Naturale, Comune di Verona, 37126 Verona, Italy

**Keywords:** *Artemisia* spp., Bioprospection, Antioxidants, Dicaffeoylquinic acids, Sesquiterpenes, Artemisinin, Metabolomics, Plant sciences

## Abstract

Species from genus *Artemisia* are widely distributed throughout temperate regions of the northern hemisphere and many cultures have a long-standing traditional use of these plants as herbal remedies, liquors, cosmetics, spices, etc. Nowadays, the discovery of new plant-derived products to be used as food supplements or drugs has been pushed by the exploitation of bioprospection approaches. Often driven by the knowledge derived from the ethnobotanical use of plants, bioprospection explores the existing biodiversity through integration of modern omics techniques with targeted bioactivity assays. In this work we set up a bioprospection plan to investigate the phytochemical diversity and the potential bioactivity of five *Artemisia* species with recognized ethnobotanical tradition (*A. absinthium*, *A. alba*, *A. annua*, *A. verlotiorum* and *A. vulgaris*), growing wild in the natural areas of the Verona province. We characterized the specialized metabolomes of the species (including sesquiterpenoids from the artemisinin biosynthesis pathway) through an LC–MS based untargeted approach and, in order to identify potential bioactive metabolites, we correlated their composition with the in vitro antioxidant activity. We propose as potential bioactive compounds several isomers of caffeoyl and feruloyl quinic acid esters (e.g. dicaffeoylquinic acids, feruloylquinic acids and caffeoylferuloylquinic acids), which strongly characterize the most antioxidant species *A. verlotiorum* and *A. annua*. Morevoer, in this study we report for the first time the occurrence of sesquiterpenoids from the artemisinin biosynthesis pathway in the species *A. alba*.

## Introduction

Bioprospection is defined as the systematic and organized exploration of natural resources and products derived from microorganisms, plants, fungi and animals, whose exploitation has the potential to generate significant economic and social benefits^1^. This approach has been applied for centuries by humans, who have always looked at plants as a crucial source of botanical drugs and other compounds for their well-being. Nowadays, about 25% of the drugs prescribed in modern medicine come from plants or are synthetic derivatives obtained from molecular plant precursors^2^, while, on the other side, a great part of the population living in developing countries still relies on traditional herbal remedies for their primary healthcare (WHO reports). However, recent literature estimates that only 15% of the species that are used for the treatment of diseases (about 70,000) have been studied for their medical use^2^, emphasizing the imperative for additional exploration. The discovery of new plant-derived drugs has classically relied on ethnobotanical information^3^, as in the case of artemisinin discovery from the herbaceous plant *Artemisia annua*^4,5^. Nonetheless, with the rise of *omics* sciences, new perspectives have emerged about the combination of ethnobotanical, phytochemical, and molecular phylogenetic data, and promising predictions of medicinal plant uses have been developed^3,6^. For instance, the improvement of DNA sequencing techniques enabled the phylogenetic reconstruction of plant clusters that include species with a prominent medicinal use and demonstrated bioactivities, allowing the acceleration for the discovery of new potential bioactive compounds in related taxa^3^.

The *Artemisia* genus (family Asteraceae) comprises about 500 species of herbs and shrubs that are either biannual, annual or perennial^7^ and that are widely distributed in temperate regions of Europe, Asia, North Africa and North America. In many cultures, several species from this genus are characterized by a long-standing ethnobotanical use including preparation of herbal remedies for treatment of major and minor ailments (e.g. fever, hypertension, diabetes and malaria), preparation of herbal teas, alcoholic beverages, tonics and cosmetics or cultivation as crops^8,9^. Many recent studies have unveiled the pleiotropic pharmacological profile of the genus reporting a broad spectrum of bioactivities reflecting the huge number and variety of phytochemicals found in each species. These include anthelmintic, antimalarial, antitubercular, antiviral, antihyperlipidemic, antiemetic, antidepressant, anticancer, anti-asthmatic, antihypertensive, antidiabetic, anxiolytic, hepatoprotective, gastroprotective, and insecticidal action^7,10–12^.

In this work we set up a bioprospection plan based on literature of local flora to investigate the phytochemical diversity and the potential bioactivity of five *Artemisia* species with recognized ethnobotanical tradition: *A. absinthium* L.*, A. alba* Turra*, A. annua* L.*, A. verlotiorum* Lamotte*, A. vulgaris* L. (Table 1). These species grow spontaneously in the natural areas within the province of Verona and within the Lessinia regional park, an area featured by hilly and mountainous environments, where the climate is classified as cold with no dry season and warm summer^13^. In this bioprospection work, we combined a sampling plan covering different growing seasons with an untargeted metabolomics approach to profile the specialized metabolomes of the five *Artemisia* species (including sesquiterpenoids from the artemisinin biosynthesis pathway) and correlate them with the in vitro antioxidant activity measured by FRAP and DPPH assays.

**Table 1:** Five selected *Artemisia* spp. used in this work with their ascribed medicinal properties.

Species	Geographic distribution	Growing season	Medicinal use/bioactivities	References
*A. absinthium* (wormwood)	Europe, Middle East, North Africa, Asia; Italy: indigenous entity	Perennial shrub	Treatment of gastrointestinal problems, anorexia, and indigestion; antiparasitic effects, pain; antispasmodic, febrifuge, stomachic, cardiac stimulant and anthelmintic effects	^14–17^
*A. alba* (white wormwood)	Europe south-east; Italy: indigenous entity	Perennial shrub	Burns, contusion, digestive antimicrobial activity	^18–20^
*A. annua* (sweet wormwood)	Asia, Europe, North Africa and North America Italy: neophyte allochthonous invasive species (Galasso et al. 2018)	Annual herb	Antimalarial, anthelmintic, antipyretic, antiseptic, antispasmodics activities	^9,14,21,22^
*A. verlotiorum* (Chinese mugwort)	Eastern Asia, South central Europe; Italy: neophyte allochthonous invasive species (Galasso et al. 2018)	Perennial herb	Treatment of hypertension; fever; psoriasis; circulatory, digestive, genito-urinary and respiratory disorders	^23,24^
*A. vulgaris* (common mugwort)	Asia, Europe, North America; Italy: indigenous entity	Perennial herb	Antioxidant, hypolipidemic, hepatoprotective, antispasmolytic, analgesic, estrogenic, cytotoxic, antibacterial, antifungal, hypotensive, and broncholytic effects	^17,25–27^

## Results and discussion

### Metabolic profiles of *Artemisia* spp. methanolic extracts

**Figure 1:**
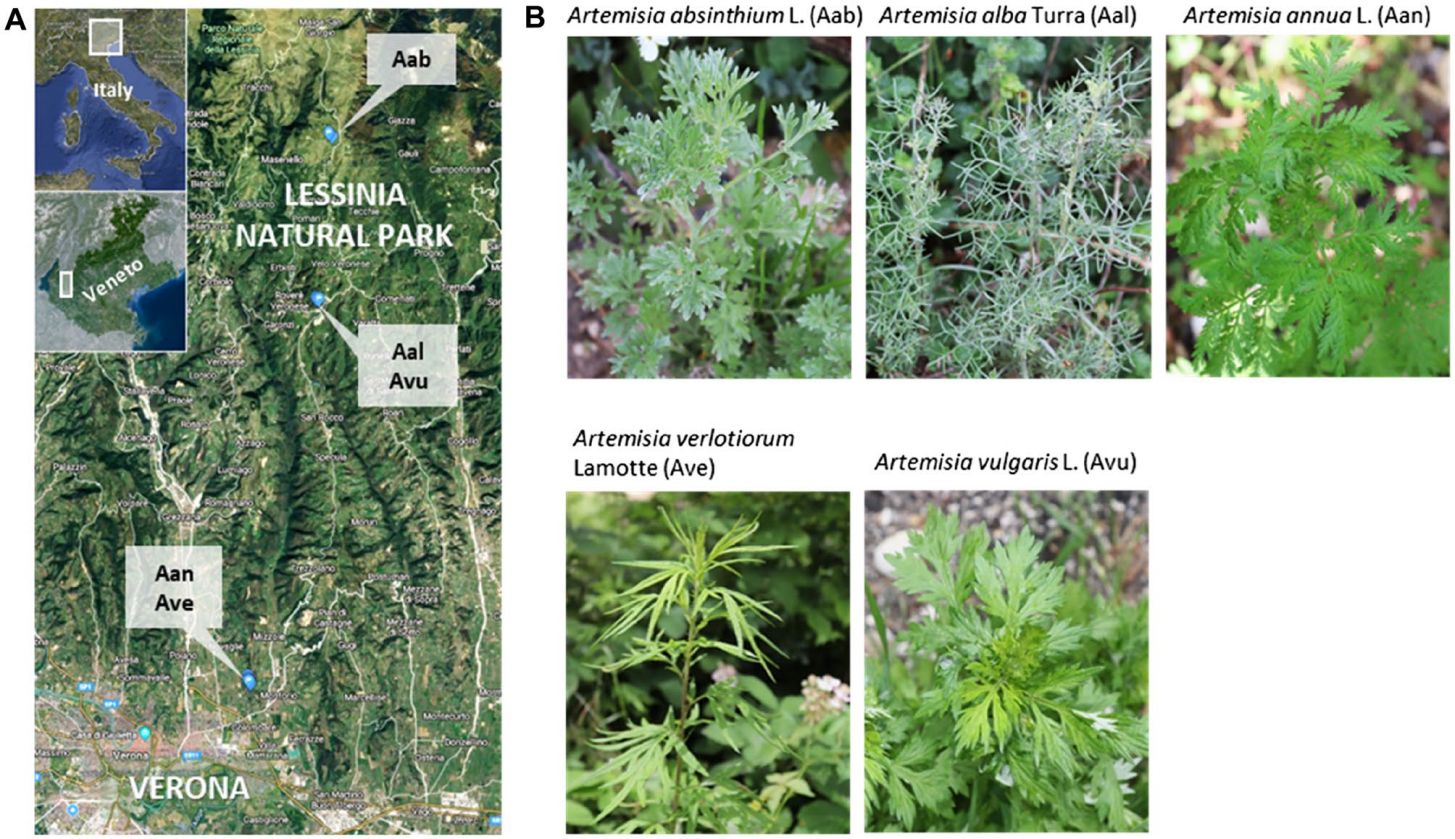
Geolocation of the sampling spots in the northern area of the Verona province (**A**) and pictures of the five *Artemisia* spp. plants collected (**B**). The maps were obtained from Google Maps (2023).

In this work, the high sensitivity and wide analytical range of an LC–MS-based untargeted approach was used to profile nonvolatile medium-polar metabolites extracted from the aerial organs of five *Artemisia* species (*A. absinthium,* Aab; *A. alba*, Aal; *A. annua*, Aan; *A. verlotiorum*, Ave; *A. vulgaris*, Avu) collected throughout three sampling seasons (2019–2021) in the natural areas around Verona (Fig. 1).

**Figure 2:**
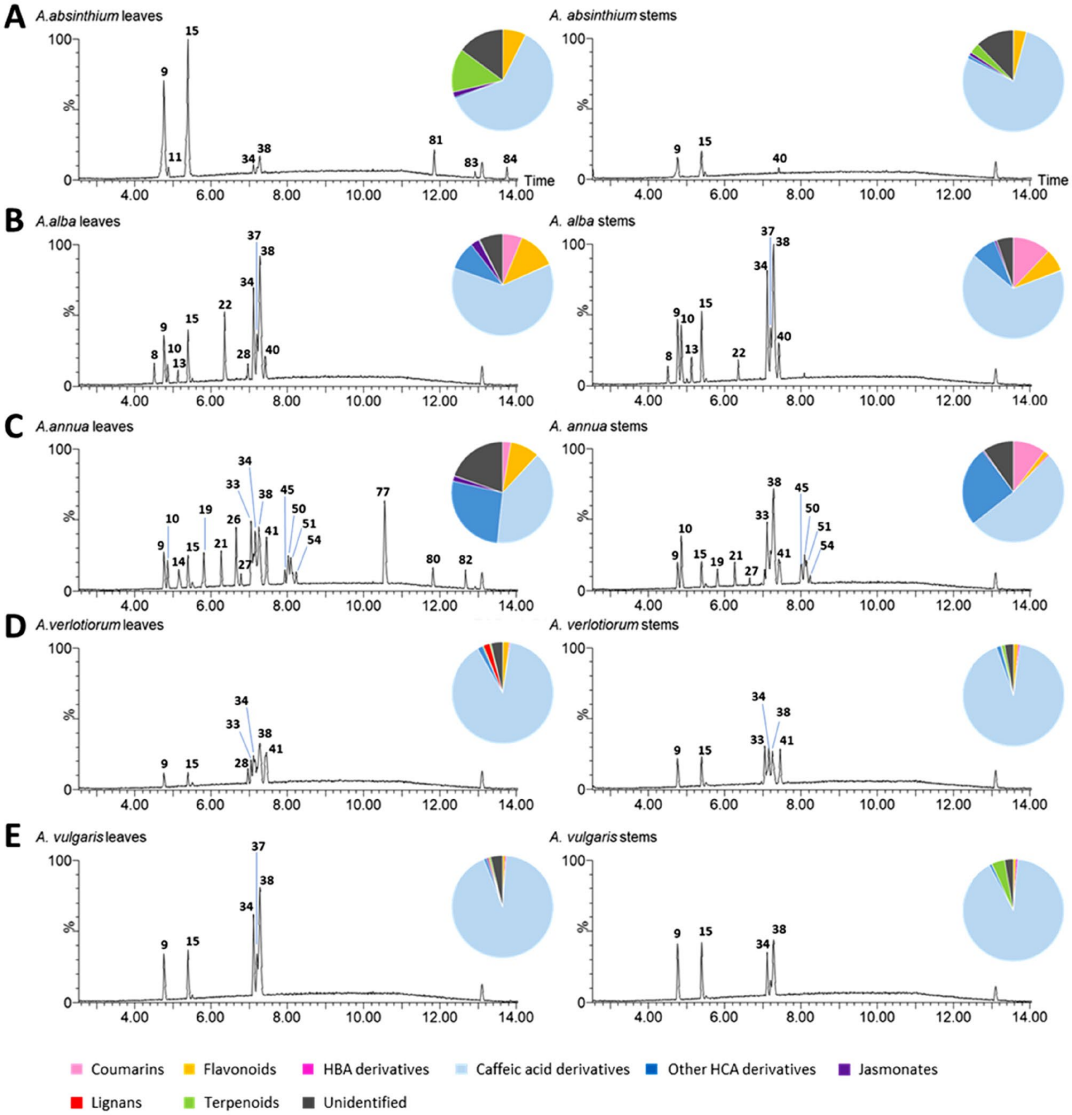
Secondary metabolomes of *Artemisia* spp. Exemplificative base peak chromatograms recorded in LC–MS–ESI^−^ (intensity scaled to 2.5 × 10^5^) of leaves (left) and stems (right) are shown together with pie charts representing the metabolite classes according to the total LC–MS signal detected. Peak annotation numbers refer to Table 2. *HBA* hydroxybenzoic acid, *HCA* hydroxycinnamic acid derivatives.

**Figure 3:**
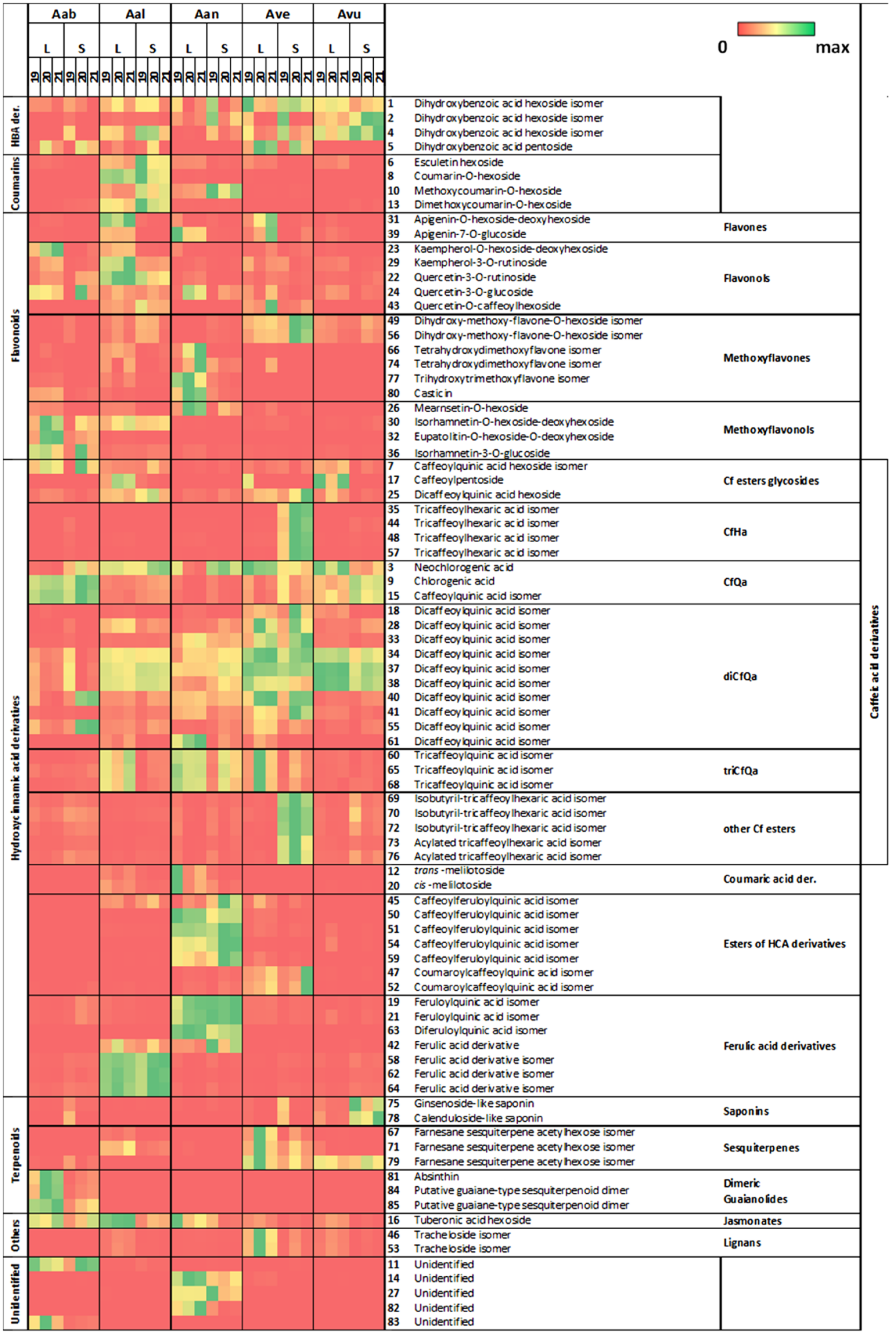
Heat map showing the average relative percentage abundance (respect to the total LC–MS signal of each metabolome) of all metabolites tentatively identified in *Artemisia* spp. Leaves (L) and stems (S) over a 3-year sampling period (2019, 2020, 2021). The metabolite identification numbers match those in the dataset. Aab, *A. absinthium*; Aal, *A. alba*; Aan, *A. annua*; Ave, *A. verlotiorum*; Avu, *A. vulgaris*. *Cf* caffeoyl, *CfQa* caffeoylquinic acids, *CfHa* caffeoylhexaric acids, *HBA* hydroxybenzoic acid, *HCA* hydroxycinnamic acid, *der* derivatives. This heatmap was created manually from the dataset with the conditional formatting tool of Microsoft Excel (v. 2401; https://www.microsoft.com/it-it/microsoft-365/excel).

Methanolic extracts of *Artemisia* spp. leaves and stems were produced and analyzed by untargeted UPLC–ESI–MS leading to the detection of 226 *m/z* features in negative ionization mode (Supplementary File 1) and to the identification of 80 secondary metabolites. The latter are listed in Table 2 together with their chromatographic and spectral features. Representative chromatographic profiles of leaf and stem methanolic extracts are shown in Fig. 2, together with pie charts depicting the metabolome composition according to the percentage of each metabolite class with respect to the total LC–MS signal detected. Moreover, to describe single compounds characterizing the various *Artemisia* species, a relative comparison of the percentages of all identified metabolites, grouped according to the respective metabolite class, is reported in the heatmap of Fig. 3.

**Table 2:** Secondary metabolites identified in *Artemisia* spp. samples by UPLC-ESI–MS^−^ analysis.

	Rt (min)	*m/z* (–) detected	Putative identification	Formula	ESI^−^ detected ion	*m/z* (–) expected	Mass error (ppm)	MS/MS	λ max (nm) UV–Vis	MSI	Reference
1	3.54	315.0716	Dihydroxybenzoic acid hexoside isomer	C_13_H_16_O_9_	[M–H]^−^	315.0710	1.766	108.022; 153.018	na	2	
2	3.92	315.0717	Dihydroxybenzoic acid hexoside isomer	C_13_H_16_O_9_	[M–H]^−^	315.0710	2.110	108.022; 153.018	na	2	
3	4.09	353.0872	Neochlorogenic acid (5-caffeoylquinic acid)	C_16_H_18_O_9_	[M–H]^−^	353.0873	− 0.072	135.044; 179.035; 191.057	245, 326	1	
4	4.27	315.0719	Dihydroxybenzoic acid hexoside isomer	C_13_H_16_O_9_	[M–H]^−^	315.0710	2.935	108.022; 153.018	na	2	
5	4.31	285.0619	Dihydroxybenzoic acid pentoside	C_12_H_14_O_8_	[M–H]^−^	285.0610	− 0.308	108.022; 153.018	na	2	
6	4.33	339.0715	Esculetin hexoside	C_15_H_16_O_9_	[M–H]^−^	339.0716	− 0.290	177.0194	na	2	^28^
7	4.41	515.1400	Caffeoylquinic acid hexoside isomer	C_22_H_28_O_14_	[M–H]^−^	515.1401	− 0.082	na	na	2	
8	4.50	369.0820	Coumarin-O-hexoside	C_15_H_16_O_8_	[M + FA–H]^−^	369.0822	− 0.412	133.024; 162.025	246, 317	2	^29^
9	4.76	353.0875	Chlorogenic acid (3-caffeoilquinic acid)	C_16_H_18_O_9_	[M–H]^−^	353.0872	− 0.073	135.044; 179.035; 191.057	242, 325	1	
10	4.86	399.0926	Methoxycoumarin-O-hexoside	C_16_H_18_O_9_	[M + FA–H] ^−^	399.0927	− 0.268	191.034; 176.010	247, 290, 336	2	
11	4.88	189.0767	Unidentified	C_8_H_14_O_5_	[M–H]^−^	189.0763	–	115.040; 99.081	na		
12	4.89	325.0923	*trans*-Melilotoside	C_15_H_18_O_8_	[M–H]^−^	325.0923	− 0.126	163.039	275, 310	2	^30^
13	5.12	429.1033	Dimethoxycoumarin-O-hexoside	C_17_H_20_O_10_	[M + FA–H]^−^	429.1033	− 0.061	221.046; 206.021	246, 293, 330	2	^31^
14	5.16	581.1511	Unidentified	C_26_H_30_O_15_	[M–H]^−^	581.1506	0.772	239.056; 209.046; 167.035; 269.067	na	4	
15	5.39	353.0876	Caffeoylquinic acid isomer	C_16_H_18_O_9_	[M–H]^−^	353.0872	− 0.115	135.044; 179.035; 191.057	243, 319	2	
16	5.40	387.1656	Tuberonic acid hexoside	C_18_H_28_O_9_	[M–H]^−^	387.1655	0.258	207.102; 163.112	na	2	^32^
17	5.52	311.0771	Caffeoylpentoside	C_14_H_16_O_8_	[M–H]^−^	311.0766	− 0.159	179.035	246, 323	2	
18	5.57	515.1184	Dicaffeoylquinic acid isomer	C_25_H_24_O_12_	[M–H]^−^	515.1189	0.095	135.044; 179.035; 191.057; 353.085		2	
19	5.81	367.1026	Feruloylquinic acid isomer	C_17_H_20_O_9_	[M–H]^−^	367.1029	− 0.932	191.057; 193.051	246, 325	2	
20	5.91	325.0923	*cis*-Melilotoside	C_15_H_18_O_8_	[M–H]^−^	325.0923	− 0.190	163.039	261	2	^30^
21	6.26	367.1028	Feruloylquinic acid isomer	C_17_H_20_O_9_	[M–H]^−^	367.1029	− 0.159	191.057; 193.051	246, 325	2	
22	6.34	609.1455	Quercetin-3-O-rutinoside	C_27_H_30_O_16_	[M–H]^−^	609.1450	0.790	300.029; 301.033	254, 348	1	
23	6.44	593.1512	Kaempherol-O-hexoside-deoxyhexoside	C_27_H_30_O_15_	[M–H]^−^	593.1506	0.974	na	na	2	
24	6.57	463.0874	Quercetin-3-O-glucoside	C_21_H_20_O_12_	[M–H]^−^	463.0876	− 0.452	300.027; 271.024; 255.029; 301.035	254, 353	1	
25	6.61	677.1730	Dicaffeoylquinic acid hexoside	C_31_H_34_O_17_	[M–H]^−^	677.1718	1.748	na	na	2	
26	6.64	493.0978	Mearnsetin-O-hexoside	C_22_H_22_O_13_	[M–H]^−^	493.0982	− 0.893	315.015; 316.022; 330.039; 331.043	253, 339	2	^33^
27	6.78	389.1082	Unidentified (hexoside)	C_15_H_20_O_9_	[M + FA–H]^−^	389.1084	− 0.637	343.104; 181.052	282, 340	4	
28	6.83	515.1187	Dicaffeoylquinic acid isomer	C_25_H_24_O_12_	[M–H]^−^	515.1189	0.044	135.044; 179.035; 191.057; 353.085	246, 326	2	
29	6.83	593.1512	Kaempherol-3-O-rutinoside	C_27_H_30_O_15_	[M–H]^−^	593.1506	0.915	285.041; 284.032; 255.027; 227.034	364, 346	1	
30	6.93	623.1610	Isorhamnetin-O-hexoside-deoxyhexoside	C_28_H_32_O_16_	[M–H]^−^	623.1612	− 0.298	315.051	357	2	
31	7.00	577.1565	Apigenin-O-hexoside-deoxyhexoside	C_27_H_30_O_14_	[M–H]^−^	577.1557	1.263	269.046	na	2	
32	7.00	653.1719	Eupatolitin-O-hexoside-O-deoxyhexoside	C_29_H_34_O_17_	[M–H]^−^	653.1718	0.181	345.083	na	2	^28^
33	7.05	515.1180	Dicaffeoylquinic acid isomer	C_25_H_24_O_12_	[M–H]^−^	515.1189	0.175	135.044; 179.035; 191.057; 353.085	244, 325	2	
34	7.11	515.1188	Dicaffeoylquinic acid isomer	C_25_H_24_O_12_	[M–H]^−^	515.1189	0.029	135.044; 179.035; 191.057; 353.085	244, 325	2	
35	7.14	695.1242	Tricaffeoylhexaric acid isomer	C_33_H_28_O_17_	[M–H]^−^	695.1248	0.092	533.083; 371.061	247, 325	2	
36	7.18	477.1028	Isorhamnetin-3-O-glucoside	C_22_H_22_O_12_	[M–H]^−^	477.1033	− 0.998	271.023; 243.029; 314.037; 299.018; 285.037; 215.031	na	1	
37	7.25	515.1180	Dicaffeoylquinic acid isomer	C_25_H_24_O_12_	[M–H]^−^	515.1189	0.175	135.044; 179.035; 191.057; 353.085	246, 326	2	
38	7.27	515.1190	Dicaffeoylquinic acid isomer	C_25_H_24_O_12_	[M–H]^−^	515.1189	− 0.021	135.044; 179.035; 191.057; 353.085	243, 326	2	
39	7.29	431.0976	Apigenin-7-O-glucoside	C_21_H_20_O_10_	[M–H]^−^	431.0970	− 0.142	268.035; 269.045	266, 332	1	
40	7.41	515.1188	Dicaffeoylquinic acid isomer	C_25_H_24_O_12_	[M–H]^−^	515.1189	0.013	135.044; 179.035; 191.057; 353.085	246, 326	2	
41	7.45	515.1187	Dicaffeoylquinic acid isomer	C_25_H_24_O_12_	[M–H]^−^	515.1189	0.048	135.044; 179.035; 191.057; 353.085		2	
42	7.47	693.2023	Ferulic acid derivative	–	[M–H]^−^	–	–	193.052; 175.038; 160.016	246, 328	2	^34^
43	7.53	625.1197	Quercetin-O-caffeoylhexoside	C_30_H_26_O_15_	[M–H]^−^	625.1193	0.568	300.029; 301.033; 463.086		2	
44	7.53	695.1261	Tricaffeoylhexaric acid isomer	C_33_H_28_O_17_	[M–H]^−^	695.1248	− 0.178	371.061; 209.031; 191.057; 191.020; 179.035; 161.022; 135.044	247, 325	2	
45	7.66	529.1340	Caffeoylferuloylquinic acid isomer	C_26_H_26_O_12_	[M–H]^−^	529.1340	0.076	191.057	246, 326	2	
46	7.66	549.1975	Tracheloside isomer	C_27_H_34_O_12_	[M–H]^−^	549.1972	0.538	387.1650; 161.025	247, 323	2	^28^
47	7.68	499.1235	Coumaroylcaffeoylquinic acid isomer	C_25_H_24_O_11_	[M–H]^−^	499.1240	0.099	191.057; 179.034; 163.042	247, 323	2	
48	7.71	695.1271	Tricaffeoylhexaric acid isomer	C_33_H_28_O_17_	[M–H]^−^	695.1248	− 0.328	371.061; 209.031; 191.057; 191.020; 179.035; 161.023; 135.044	247, 325	2	
49	7.73	461.1084	Dihydroxy-methoxy-flavone-O-hexoside isomer	C_22_H_22_O_11_	[M–H]^−^	461.1080	− 0.094	na	na	2	
50	7.92	529.1343	Caffeoylferuloylquinic acid isomer	C_26_H_26_O_12_	[M–H]^−^	529.1346	− 0.551	191.057	246, 326	2	
51	8.02	529.1343	Caffeoylferuloylquinic acid isomer	C_26_H_26_O_12_	[M–H]^−^	529.1346	− 0.551	191.057	246, 326	2	
52	8.05	499.1238	Coumaroylcaffeoylquinic acid isomer	C_25_H_24_O_11_	[M–H]^−^	499.1240	0.038	191.057; 179.035; 163.042	247, 323	2	
53	8.05	549.1972	Tracheloside isomer	C_27_H_34_O_12_	[M–H]^−^	549.1972	− 0.053	na	na	2	
54	8.09	529.1350	Caffeoylferuloylquinic acid isomer	C_26_H_26_O_12_	[M–H]^−^	529.1340	1.951	191.057	246, 326	2	
55	8.09	515.1186	Dicaffeoylquinic acid isomer	C_25_H_24_O_12_	[M–H]^−^	515.1189	0.053	135.044; 179.035; 191.057; 353.085	246, 326	2	
56	8.23	461.1083	Dihydroxy-methoxy-flavone-O-hexoside isomer	C_22_H_22_O_11_	[M–H]^−^	461.1080	− 0.065	na	na	2	
57	8.37	695.1267	Tricaffeoylhexaric acid isomer	C_33_H_28_O_17_	[M–H]^−^	695.1248	− 0.270	371.061; 209.031; 191.057; 191.020; 179.035; 161.022; 135.044	247, 325	2	
58	8.58	735.2127	Ferulic acid derivative isomer	–	[M–H]^−^	–	–	175.040; 193.051; 160.016; 539.137	247, 329	2	
59	8.77	529.1344	Caffeoylferuloylquinic acid isomer	C_26_H_26_O_12_	[M–H]^−^	529.1340	0.795	191.057	246, 326	2	
60	8.78	677.1507	Tricaffeoylquinic acid isomer	C_34_H_30_O_15_	[M–H]^−^	677.1500	− 0.103	191.057; 515.118	248, 329	2	
61	8.82	515.1186	Dicaffeoylquinic acid isomer	C_25_H_24_O_12_	[M–H]^−^	515.1189	− 0.671	135.044; 179.035; 191.057; 353.085	246, 326	2	
62	8.82	735.2133	Ferulic acid derivative isomer	–	[M–H]^−^	–	–	175.040; 193.051; 160.016; 539.137	247, 329	3	
63	8.88	543.1500	Diferuloylquinic acid isomer	C_27_H_28_O_12_	[M–H]^−^	543.1502	− 0.477	191.057; 193.051	249, 327	2	
64	8.97	735.2124	Ferulic acid derivative isomer	–	[M–H]^−^	–	–	175.040; 193.051; 160.016; 539.137	247, 329	3	
65	9.04	677.1509	Tricaffeoylquinic acid isomer	C_34_H_30_O_15_	[M–H]^−^	677.1500	1.382	191.057; 515.118	na	2	
66	9.26	345.0612	Tetrahydroxydimethoxyflavone isomer	C_17_H_14_O_8_	[M–H]^−^	345.0610	0.518	327.012; 315.015	250, 340	2	^28^
67	9.34	487.2530	Farnesane sesquiterpene acetylhexose isomer	C_23_H_38_O_8_	[M + FA–H]^−^	487.2543	− 2.66	441.248; 221.066; 161.045; 149.047; 131.032; 113.028; 101.026	na	4	
68	9.36	677.1530	Tricaffeoylquinic acid isomer	C_34_H_30_O_15_	[M–H]^−^	677.1500	4.427	191.057; 515.118	na	2	
69	9.45	765.1673	Isobutyril-tricaffeoylhexaric acid isomer	C_37_H_34_O_18_	[M–H]^−^	765.1660	− 0.17	279.071; 441.104; 603.137; 191.023		2	
70	9.70	765.1702	Isobutyril-tricaffeoylhexaric acid isomer	C_37_H_34_O_18_	[M–H]^−^	765.1660	− 0.55	279.071; 441.104; 603.137; 191.023		2	^35^
71	9.76	487.2532	Farnesane sesquiterpene acetylhexose isomer	C_23_H_38_O_8_	[M + FA–H]^−^	487.2543	− 2.29	441.248; 221.066; 161.045; 149.047; 131.032; 113.028; 101.026	248, 341	4	
72	9.92	765.1683	Isobutyril-tricaffeoylhexaric acid isomer	C_37_H_34_O_18_	[M–H]^−^	765.1660	− 0.31	279.071; 441.104; 603.137; 191.023		2	^35^
73	10.05	779.1798	Acylated tricaffeoylhexaric acid isomer	C_38_H_36_O_18_	[M–H]^−^	779.1820	0.28	293.087; 455.118; 617.148		3	^35^
74	10.10	345.0606	Tetrahydroxydimethoxyflavone isomer	C_17_H_14_O_8_	[M–H]^−^	345.0610	− 1.266	na	na	2	^28^
75	10.48	955.4918	Ginsenoside-like saponin	C_48_H_76_O_19_	[M–H]^−^	955.4902	1.605	793.441; 731.434; 613.374; 569.381; 523.377	na	3	^36^
76	10.51	779.1781	Acylated tricaffeoylhexaric acid isomer	C_38_H_36_O_18_	[M–H]^−^	779.1820	0.50	293.087; 455.118; 617.148		3	^35^
77	10.54	359.0765	Trihydroxytrimethoxyflavone isomer	C_18_H_15_O_8_	[M–H]^−^	359.0767	− 0.518	286.013; 344.053; 329.030; 314.007; 258.017; 230.022; 202.027	250, 350	2	^37^
78	11.10	793.4369	Calenduloside-like saponin	C_42_H_66_O_14_	[M–H]^−^	793.4374	− 0.702	631.381; 613.374; 569.386; 455.354	na	3	^38^
79	11.35	487.2536	Farnesane sesquiterpene acetylhexose isomer	C_23_H_38_O_8_	[M + FA–H]^−^	487.2543	− 1.458	441.248; 221.066; 161.045; 149.047; 131.032; 113.028; 101.026	na	4	
80	11.83	373.0923	Casticin	C_19_H_18_O_8_	[M–H]^−^	373.0923	− 0.202	358.071; 343.043; 285.003; 257.009; 229.015	254, 350	2	^28^
81	11.85	541.2799	Absinthin	C_30_H_40_O_6_	[M + FA–H]^−^	541.2801	− 0.404	495.272; 451.284; 433.274; 337.804; 293.820	251, 353	1	
82	12.66	265.1443	Unidentified	C_15_H_22_O_4_	[M–H]^−^	265.1440	1.146	233.115; 189.126; 177.093	na	4	
83	12.92	571.2543	Unidentified	–	–	–	–	na	na		
84	13.76	539.2639	Putative guaiane-type sesquiterpenoid dimer	C_30_H_38_O_6_	[M + FA–H]^−^	539.2645	− 1.113	493.269; 471.232; 293.105;	251, 353	4	^39^
85	14.86	507.2379	Putative guaiane-type sesquiterpenoid dimer	C_30_H_36_O_7_	[M–H]^−^	507.2383	− 0.79	463.246; 445.243; 259.099; 215.108	na	4	^40^

Peak IDs refer to peak numbers represented in Fig. 2.

Identification level was established according to metabolomics standards initiative (MSI)^41^: unambiguous identifications (level 1), comparison with reference standards analyzed under equal experimental conditions; putative assignments (level 2), MS data similarity with literature data or public databases; level 3 was established by spectral similarity to chemical class of compounds and chemotaxonomic data when no literature/database data are available for proposed structures, level 4 unidentified.

*Rt* retention time, *MS/MS* diagnostic fragments detected, *na* not available.

The main specialized metabolites composing the metabolomes of *Artemisia* spp. comprise derivatives of hydroxycinnamic acid (HCA), mainly caffeic and ferulic acid differentially esterified with one or multiple organic acid moieties, O-glycosylated flavonoids (mainly flavonols and flavones together with their O-methylated derivatives), glycosylated coumarins and a few other metabolites identified as glycosides of hydroxybenzoic acids, lignans, saponins and sesquiterpenes. In general, a high level of metabolome characterization was achieved, resulting in a metabolite annotation range of 85–97% of total LC–MS signal, with *A. annua* and *A. abisnthium* being the species with the higher proportion of unidentified metabolites left. The HCA derivatives represented the most abundant metabolite class, ranging from 62 to 94% of total LC–MS signal in *A. absinthium* and *A. verlotiorum*, respectively. As can be observed in the chromatograms of Fig. 2, in all five species this class was dominated by the caffeic acid esters of quinic acid, in particular two caffeoylquinic acid isomers (compounds **9**, **15**) and many isomers of dicaffeoylquinic acid (**28**, **33**, **34**, **37**, **38**, **40**, **41**). The prevalence of caffeic acid derivatives in the *Artemisia* metabolome is well pronounced in *A. absinthium, A. verlotiorum* and *A. vulgaris,* in which they represent the main peaks*,* whereas a more diversified profile is observed in favor of other compound classes in *A.alba* and *A,annua* (e.g., other HCA derivatives, coumarins and flavonoids).

A detailed description of the metabolic profiles of leaves and stems of the five *Artemisia* species is reported below according to data presented in Figs. 2 and 3.

*A. absinthium* (Fig. 2A) leaves are mainly characterized by the presence of two isomers (**9**, **15**) of caffeoylquinic acid, one of them identified as 3-O-caffeoylquini acid (chlorogenic acid; **15**) and of two abundant isomers of dicaffeoylquinic acid (**34**, **38)**; also caffeoylquinic acid hexoside was best represented in this species (**7**). The leaves of *A. absinthium*, compared to the other species, present higher proportion of the flavonols kaempferol-O-hexoside-deoxyhexoside (**23**), isorhamnetin-O-hexoside-deoxyhexoside (**30**), eupatolitin-O-deoxyhexoside-O-hexoside (**32**) and isorhamnetin-3-O-glucoside (**36**). Moreover, in leaves and, at a lower level, in stems of this species only, we detected high amounts of the dimeric guaianolide absinthin (**81**), which is described as a specific marker of *A. absinthium* and is responsible for the bitterness of absinth^7,28,42^. In addition, two other compounds annotated as guaiane-type sesquiterpene dimers (**84**, **85**) were putatively identified according to recent literature data and in silico fragmentation analysis^39,40^. The stems of *A. absinthium* showed a much more simplified profile, including the same caffeoylquinic acid isomers found in the leaves together with two less represented dicaffeoylquinic acid isomers (**40**, **55**). Furthermore, in both leaves and stems we observed two unidentified metabolites with a molecular ion of 189.07 *m/z* (**11)** and 571.25 *m/z* (**83**) characterized, respectively, by higher and lower polarity.

Leaves and stems of *A. alba* (Fig. 2B) were strongly characterized by the glycosides of different coumarins including esculetin hexoside (**6**), coumarin-O-hexoside (**8**), methoxycoumarin-O-hexoside (**10**) and dimethoxycoumarin-O-hexoside (**13**). These organs present major peaks attributed to caffeoylquinic acids (**9**, **15**) and various isomers of dicaffeoylquinic acid (**28**, **34**, **37**, **38**, **40**). Less represented hydroxycinnamic acid derivatives include dicaffeoylquinic acid hexoside (**25**), caffeoylpentoside (**17**) and three isomers of tricaffeoylquinic acid (**60**, **65**, **68**), which were mostly abundant in the leaves. Moreover, this species is strongly characterized by the presence of four ferulic acid derivatives, one with a molecular ion of 693.20 *m/z* (**42)** and three isomers showing a molecular ion of 735.21 *m/z* (**58**, **62**, **64**,); the resulting neutral loss of 42.01 Da and the higher retention time strongly suggest that the latter could be the acetylated forms of compound **42**. With respect to stems**,** *A. alba* leaves presented higher levels of one isomer of a dihydroxybenzoic acid hexoside (**4**) and various flavonoids glycosides including quercetin-3-O-rutinoside (**22**), kaempferol-O-hexoside-deoxyhexoside (**23**), kaempferol-3-O-rutinoside (**29**), isorhamnetin-O-hexoside-deoxyhexoside (**30**) and apigenin-O-hexoside-deoxyhexoside (**31**). Interestingly, according to literature^32^, we detected high levels in the leaves of *A. alba* of tuberonic acid hexoside (**16**), which belong to the class of jasmonate glycosylated derivatives, i.e. phospholipid-derived hormones that regulate plant development and responses to environmental stress.

*A. annua* (Fig. 2C) was the species presenting the most diversified profile of its secondary metabolome, being characterized by many major compounds belonging to different metabolite classes. In general, the highest diversification was observed within the class of hydroxycinnamic acid derivatives and their esters. In particular, two isomers (**12**, **20**) of *o*-coumaric acid glucoside (*trans*- and *cis*-melilotoside), previously reported in *A. annua* tea infusions^30^, were detected at high levels in the leaves together with other hydroxycinnamate esters that include feruloyl moieties, such as two feruloylquinic acid isomers (**19**, **21**), one diferuloylquinic acid isomer (**63**) and five caffeoylferuloylquinic acid isomers, one characterizing mostly the leaves (**50**) and the other ones the stems (**45**, **51**, **54**, **59**). Also, caffeoyl-(**9**, **15**), dicaffeoyl-(**33**, **34**, **37**, **38**, **40, 61**) and tricaffeoyl-(**60**, **65**, **68**) quinic acids were well represented in both leaves and stems within this species. Among the coumarins, the stems reported the highest levels of methoxycoumarin-O-hexoside (**10**). Leaves, on the other hand, were characterized by high amounts of flavonoids, especially methoxylated and often in aglycone form, such as casticin (**80**), which is described as a marker of *A. annua*^28^ and a trihydroxytrimethoxyflavone isomer (**77**). The latter, together with the methoxylated flavonol mearnsetin-O-hexoside (**26**), represented the major flavonoid peaks of the *A. annua* leaf chromatograms. Other characteristic flavonoids included two isomers of a tetrahydroxydimethoxyflavone (**66**, **74**), quercetin-3-O-glucoside (**24**) and apigenin-7-O-glucoside (**39**).

Finally, three unidentified metabolites (**14**, **27**, **82**) were detected at high levels in *A. annua*. In particular, compound **27** forms a molecular adduct with formic acid at 389.11 *m/z* under negative ionization conditions; its fragmentation results in the molecular ion at 343.10 *m/z* and in the fragment 181.05 *m/z,* which is indicative of the neutral loss of a hexose (− 162.05 Da). Moreover, the UV–Vis absorbance at 282 and 340 nm together with the fact that it is detected as formic acid adduct, supports the hypothesis that this metabolite could belong to the class of coumarins.

*A. verlotiorum* (Fig. 2D) and *A. vulgaris* (Fig. 2E) presented much simpler profiles respect to the other species.

*A. verlotiorum* was distinct from all the other species for the higher content of two lignans (tracheloside isomers; **46**, **53**) and three compounds that were putatively annotated as farnesane sesquiterpene acetyl-hexose isomers according to their fragmentation pattern (**67**, **71**, **79**). Another peculiarity of this species was the high relative levels of various tricaffeoylhexaric acid isomers (**35**, **44**, **48**, **57**), including those acylated to isobutyric acid moieties commonly found in other Asteraceae species (**69**, **70**, **72)**;^35^ and other tricaffeoyl acid esters (**73**, **76**) that were particularly high in the stems. The leaves and stems were also characterized by many different isomers of dicaffeoyl (**18**, **28**, **33**, **34**, **37**, **38**, **40**, **41**, **55**) and tricaffeoyl (**60**, **65**, **68**) quinic acid, by dicaffeoylquinic acid hexoside (**25**) and coumaroylcaffeoylquinic acid isomers (**47**, **52**), while neochlorogenic acid (**3**) levels were higher in the leaves of this species. Other characteristic metabolites of *A. verlotiorum* leaves were represented by hexose (**1**) and pentose (**5**) esters of a dihydroxybenzoic acid, by quercetin-O-caffeoylhexoside (**43**) and by four flavone glycosides, which included apigenin-O-hexoside-deoxyhexoside (**31**), apigenin-7-O-glucoside (**39**) and two dihydroxymethoxyflavone-O-hexoside isomers (**49**, **56**), the latter best represented in the stems.

*A. vulgaris*, within the five species, contained lower levels of flavonoids but its stems were characterized by the presence of ginsenoside-like (**75**) and calenduloside-like (**78**) saponins. Moreover, leaves were characterized by three dicaffeoylquinic acid isomers (**34**, **37**, **38**), which were much more represented in this species respect to other ones, yet at lower amounts. In proportion, also neochlorogenic acid (**3**), caffeoyl pentoside (**17**), two dihydroxybenzoic acid hexosides (**2**,** 4**) and one sesquiterpene diol monohexoside derivative (**79**) characterize *A. vulgaris* aerial organs.

The HCA derivatives, in particular the esters of caffeic acid, were the most characterizing compounds in all the five *Artemisia* species considered in this work. All these metabolites showed accumulation patterns similar to those already reported in literature for several *Artemisia* species by using similar extraction methods and identified with different techniques from LC–MS such as NMR and HPLC–DAD^7,14,43^. The degree and the nature of esterification determines the type of the specific ester isomer that is produced and, for several HCA derivatives, we observed species-specific esterification patterns. This indicates a diversification in the late enzyme of the pathway, among *Artemisia* spp., such those involving the esterases^44^. It is interesting to notice how distribution of isomers of different esters of caffeic acid changes also between different organs (stems or leaves) of each species. For instance, various tricaffeoylhexaric acid isomers, a molecule that has been already detected in various Asteraceae^45,46^, were found at high levels in *A. verlotiorum* stems, while tricaffeoylquinic acids were located mostly in the leaves. This is probably due to a different spatial distribution in the enzyme involved in the esterification of HCA biosynthesis between the different organs in each species.

As already reported by the literature, flavonoids are another widely represented class of metabolites in the *Artemisia* genus^10,42,43^. Ubiquitous presence of different glycosylated and methoxy-glycosylated flavonoids among all the five species investigated has been observed. Interestingly, in *A. annua,* and to a lesser extent also in *A. absinthium* and *A. alba,* aglycone form of different flavonoids, including the methoxylated flavonol casticin, were observed. Within plant cells, most flavonoids are present as O- or C-glycosides^47,48^, while the aglycones are more typical of extracellular exudates^49^. The presence of different flavonoid aglycones has been reported in plant exudates of different *Artemisia* species^50,51^. For instance, in *A. absinthium, A. alba* and *A. vulgaris,* different polymethoxylated flavonoid aglycones were found on the surface of aerial parts, in the extracellular environment, predominantly in leaves and floral buddings, where they probably have protective roles and/or allelopathic functions^12,52^. The occurrence of these compounds is probably related to the presence on the leaf surface of epidermal trichomes, which can synthetize and store large quantities of specialized metabolites^53^. Trichomes are epidermis appendages and can be divided into glandular trichomes (GTs) and non-glandular (NGTs) according to their morphology^54^. In particular, glandular trichomes can synthesize, store, and secrete large amounts of exudates, including alkaloids, polysaccharides, terpenoids, polyphenols, organic acids, and defensive proteins. In turn, these exudates can entrap or poison herbivores and prevent pathogen infection^55,56^. Usually Asteraceae, harbors mainly GTs, where high-value secondary metabolites, including artemisinin in *A. annua,* are produced and then stored, ready to be used in plant defensive mechanism against both biotic and abiotic stress^54,57^. Presence of glandular trichomes, has been reported for all the species investigated in this work, and can explain the observation of the above mentioned flavonoid aglycones in our samples^18,58^*.*

Considering the three different growing seasons, the strongest differences were observed for *A. annua* and *A. verlotiorum*. In *A. annua*, the relative levels of many flavonoids and hydroxycinnamic acid derivatives during 2019 was lower than 2020 and 2021. In *A. verlotiorum*, some flavonoids and various hydroxycinnamic acid derivatives showed higher relative level in 2021 compared with 2019 and 2020. This variation could be expected for herbaceous annual species^14^, such as *A. annua*, if we consider that the sampling of potentially distinct individuals over 3 years could have increased the genotypic variability of the samples. It is not excluded that some *Artemisia* species are more sensitive to environmental conditions than others and modulate the levels of single or groups of metabolites in response to different stimuli. However, the type of experimental design that we adopted in this work does not allow us to precisely dissect the effects of specific climate or geographic conditions and, thus, is not suitable to investigate such complex environment-metabolome interactions.

### Antioxidant assays of *Artemisia* spp. methanolic extracts

In this work we performed in vitro antioxidant assays as low cost and easy to use high-throughput screening systems for the identification of potential sources of antioxidants^59,60^. These should then be followed by confirmatory in vivo biological tests with simulated digestion samples^61^ to assess the antioxidant activity in a more physiological context. It is commonly accepted that antioxidant activity must not be tested on the basis of a single method^62^ given the involvement of different antioxidant mechanisms by the molecules present in a phytocomplex. Thus, we used FRAP and DPPH to assess the reducing capacity and radical scavenging activity, respectively, of leaf and stem methanolic extracts of the five *Artemisia* species.

**Figure 4:**
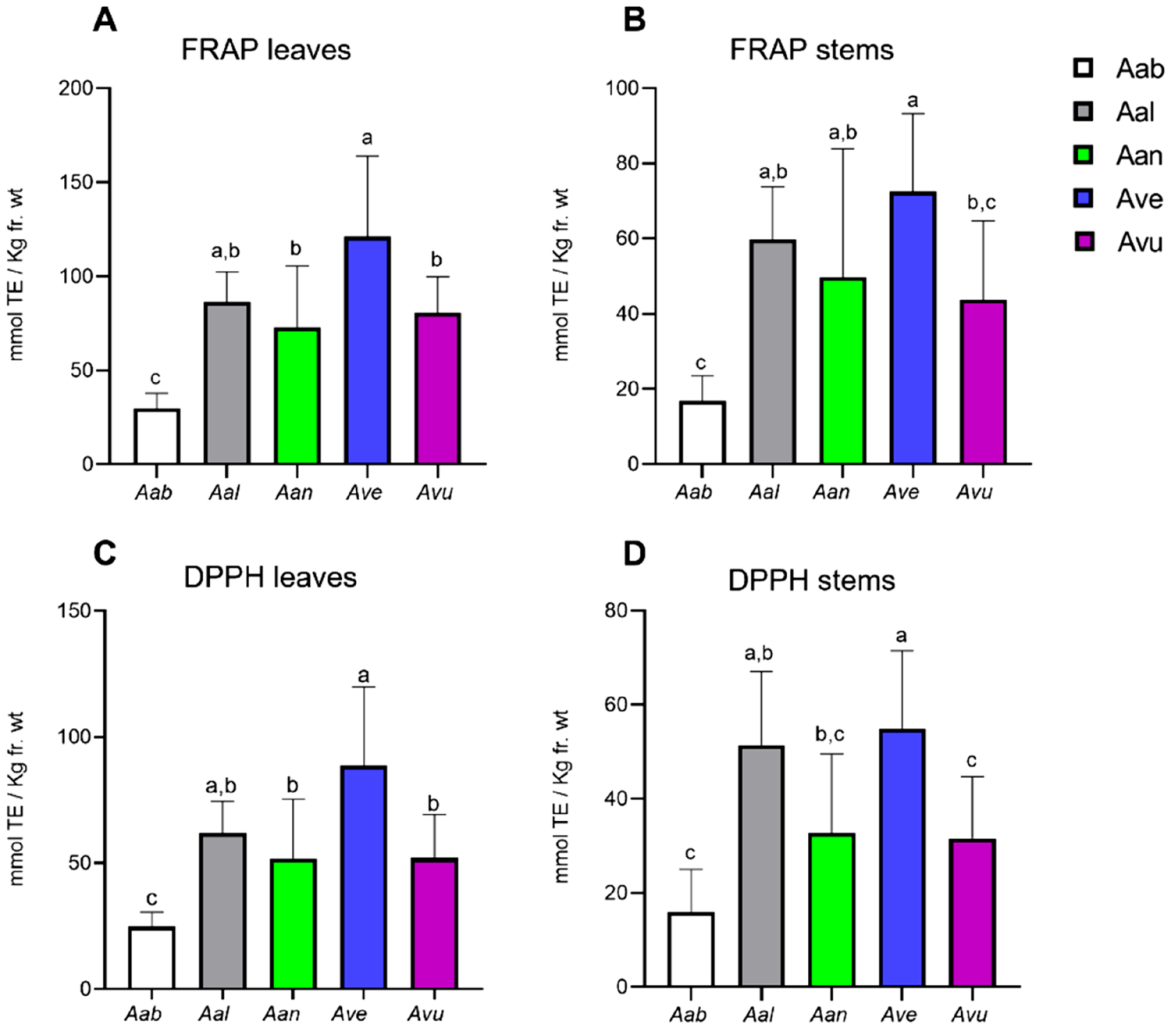
Antioxidant activity of extracts from *Artemisia* spp. leaves and stems, sampled in three independent growing seasons, and determined by FRAP (**A**, **B**) and DPPH (**C**, **D**) assays, and expressed as Trolox Equivalent Antioxidant Capacity (TEAC), in millimoles of Trolox Equivalents/kg of tissue (leaves or stem), fr. wt. Values are expressed as mean +/− standard deviation (n = 9)*.* Significant differences were calculated with one-way ANOVA.

In general, extracts from leaves showed higher antioxidant capacity compared with extracts from stems in both assays (Fig. 4).

Within the five species, *A. absinthium* showed the lowest antioxidant activity (up to 30.0 and 24.8 mmolTE/Kg fr. wt in FRAP and DPPH), while *A. verlotiorum* showed the highest antioxidant power for both FRAP (121.2 mmolTE/Kg fr. wt) and DPPH (up 88.7 mmolTE/Kg fr.wt), about four times higher than *A. absinthium*. In between these two species, *A. annua*, *A. alba* and *A. vulgaris* displayed a medium to high antioxidant activity (see Supplementary Table 1).

Many studies report the antioxidant activity of different *Artemisia* spp. extracts assayed with a broad panel of methods but a comparison with the results presented in this work is challenged by non-homogeneous expression of data (e.g. TEAC, IC_50_*,* percentage of radical scavenging, etc.) or the use of reference compounds other than Trolox. A few recent studies report the antioxidant activity of methanolic extracts of various *Artemisia* species in comparison to Trolox^19,28,63^ (Supplementary Table 2). Our results are in line with the trend observed by Trifan and colleagues for FRAP assay, in which *A. absinthium* displayed the lowest antioxidant activity. On the other hand, we did not observe higher antioxidant activity for *A. vulgaris*, as reported by the authors. In general, the TEAC values reported in all these studies for the five *Artemisia* species are five to ten times higher than our results, but this is justified by the fact that dried instead of fresh plant material was used to produce the extracts, thus resulting in higher concentrations of antioxidant compounds.

**Figure 5:**
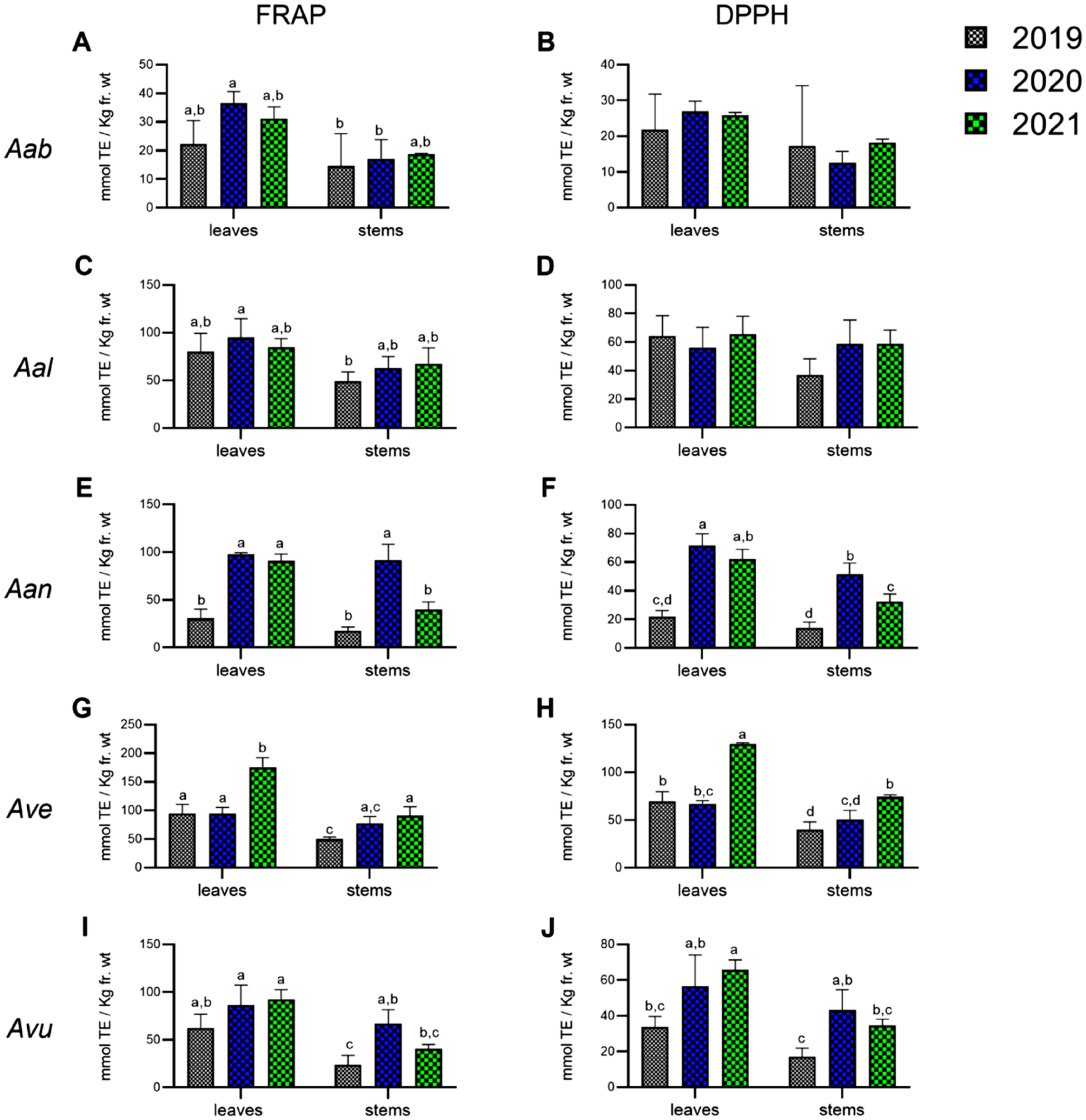
Antioxidant activity of extracts from *Artemisia* spp. leaves and stems, sampled in three independent growing seasons, and determined by FRAP (**A**, **C**, **G**, **I**) and DPPH (**B**, **D**, **F**, **H**, **J**) assays, and expressed as Trolox Equivalent Antioxidant Capacity (TEAC), in millimoles of Trolox Equivalents/kg of tissue (leaves or stem), fresh weight. Values are expressed as mean +/− standard deviation (n = 9)*.* Aab, *absinthium;* Aal, *alba;* Aan, *annua;* Ave, *verlotiorum;* Avu, *vulgaris*. Significant differences calculated with two-way ANOVA.

In Fig. 5 the antioxidant activity in each of the 3 years of sampling is shown (see also Supplementary Table 1). In some cases, a clear impact of the specific growing season on antioxidant activity was observed. For example, *A. verlotiorum* extracts showed higher antioxidant activities in 2021 than 2019 and 2020 in both FRAP (Fig. 5G) and DPPH (Fig. 5H), while *A. annua* and *A. vulgaris* showed lower antioxidant activities in 2019 compared with 2020 and 2021 (Fig. 5E, F, I, L). On the other hand, the antioxidant activity of the leaves of *A. absinthium* (Fig. 5A, B) and *A. alba* (Fig. 5C, D), did not vary significantly throughout the 3 years. According to these data, the antioxidant activity of species like *A. absinthium* and *A. alba* seems to be less influenced by the growing season as it occurs in the case of *A. annua*, *A. vulgaris* and *A. verlotiorum*.

### Correlation analysis of antioxidant data and metabolic profiles of *Artemisia* spp.

**Figure 6:**
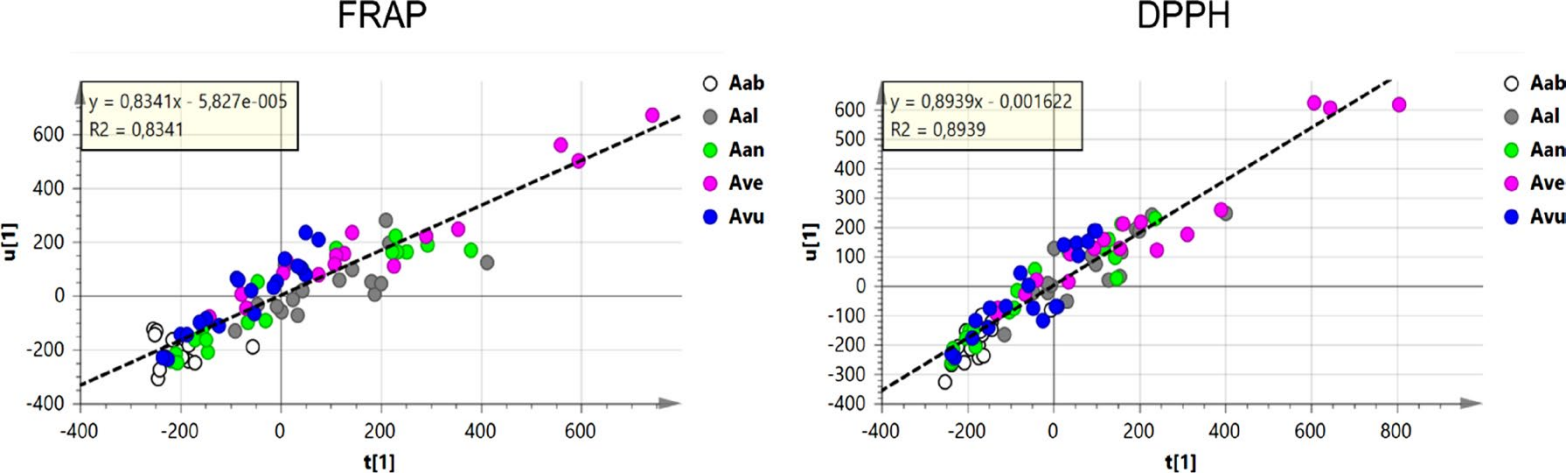
Scatter plot of OPLS analysis that correlates antioxidant activity (u) (FRAP on the left and DPPH on the right) with metabolic composition (t). Samples are colored according to the species.

In order to obtain information on which metabolites may be responsible for antioxidant activity of *Artemisia* spp. methanolic extracts, a statistical tool of multivariate analysis (OPLS) was used to find linear relations between the metabolite levels (whose *m/z* features were assigned as X variables) and the antioxidant capacity (whose mmolTE/Kg were assigned as Y variables). The score scatter plots of Fig. 6 show a good, yet not too strong, linear correlation between the metabolite levels (t, x axis) and the antioxidant activity (u, y axis), for both FRAP and DPPH (0.86 < R^2^ < 0.90), thus recalling the need for an independent OPLS analysis in each different species; this is expected, since different set of metabolites could be responsible for the overall antioxidant activity of each species. In this analysis, samples that displayed the highest mmol TE/Kg of fresh plant material clustered on the top right corner of the graph and those with the lower values in the left-down corner.

**Figure 7:**
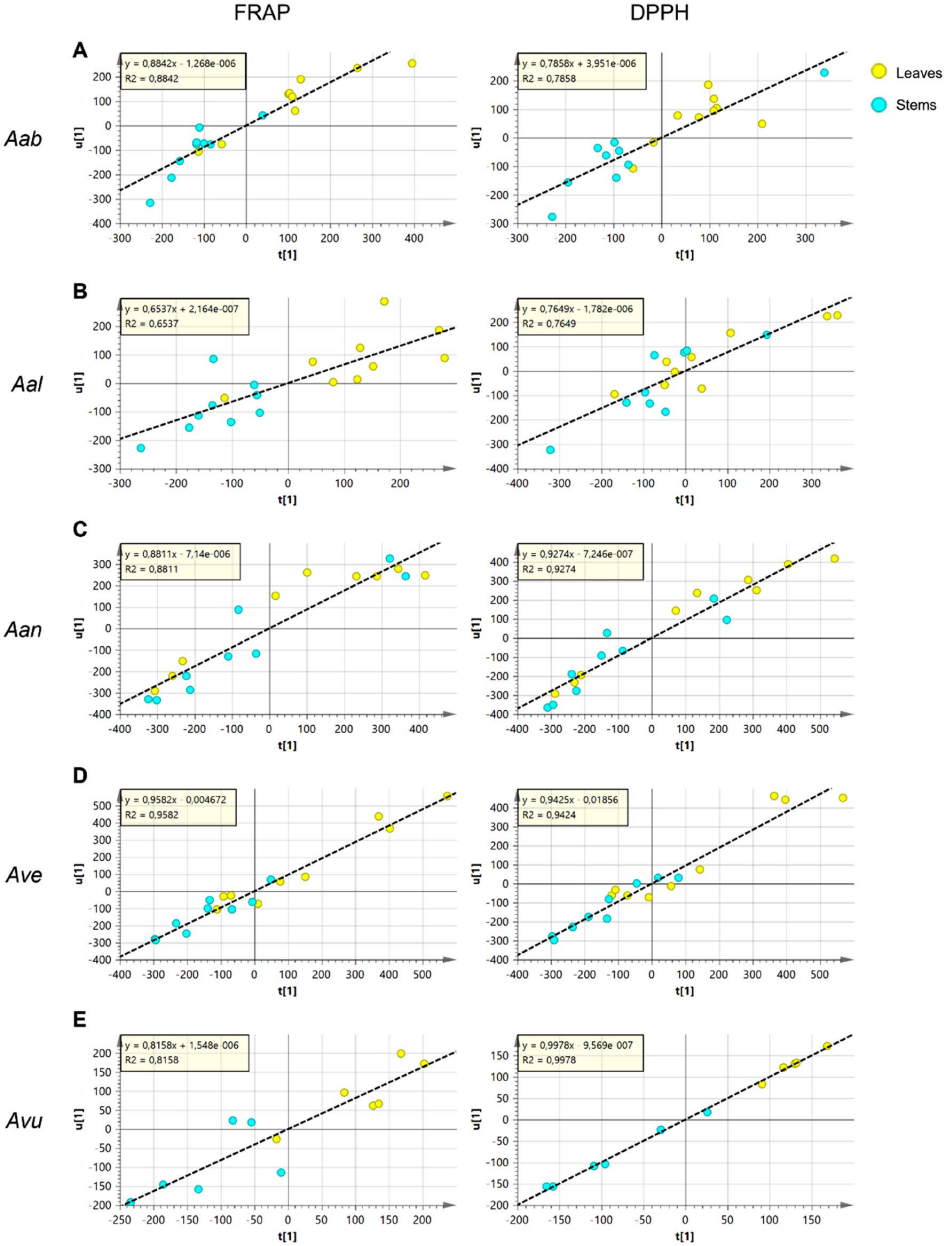
Scatter plot of OPLS analysis that correlates antioxidant activity (u) (FRAP on the left and DPPH on the right) with metabolic composition (t). Samples are colored according to the organs. (**A**) *A. absinthium* (Aab); (**B**) *A. alba* (Aal); (**C**) *A. annua* (Aan); (**D**) *A. verlotiorum* (Ave); (**E**) *A. vulgaris* (Avu).

The same analysis was applied to each of the individual species. The OPLS individual models for leaves and stem extracts of each of the species are shown in Fig. 7. The *loadings* of these OPLS analyses can be used to evaluate the contribution of each *m/z* feature, *i.e.* of each detected metabolite, to the observed antioxidant activity (Tables 3, 4).

**Table 3:** Lists of metabolites that correlate with FRAP activity in *Artemisia* spp. samples.

Species	ID	FRAP-correlating metabolites	pq(corr)
Aab	15	Caffeoylquinic acid isomer	0.96
	9	Chlorogenic acid	0.95
	81	Absinthin	0.94
	29	Kaempherol-3-O-rutinoside	0.93
	80	Casticin	0.91
	77	Trihydroxytrimethoxyflavone	0.89
	23	Kaempherol-O-hexoside-deoxyhexoside	0.89
	85	Putative guaiane-type sesquiterpenoid dimer	0.88
	30	Isorhamnetin-O-hexoside-deoxyhexoside	0.87
	84	Putative guaiane-type sesquiterpenoid dimer	0.87
Aal	22	Quercetin-3-O-rutinoside	0.91
	23	Kaempherol-O-hexoside-deoxyhexoside	0.88
	29	Kaempherol-3-O-rutinoside	0.87
Aan	37	Dicaffeoylquinic acid isomer	0.99
	51	Caffeoylferuloylquinic acid isomer	0.98
	34	Dicaffeoylquinic acid isomer	0.98
	21	Feruloylquinic acid isomer	0.96
	15	Caffeoylquinic acid isomer	0.95
	19	Feruloylquinic acid isomer	0.95
	40	Dicaffeoylquinic acid isomer	0.94
	9	Chlorogenic acid	0.94
	65	Tricaffeoylquinic acid isomer	0.91
	38	Dicaffeoylquinic acid isomer	0.91
	50	Caffeoylferuloylquinic acid isomer	0.91
	60	Tricaffeoylquinic acid isomer	0.89
	63	Diferuloylquinic acid isomer	0.88
Ave	34	Dicaffeoylquinic acid isomer	1.00
	37	Dicaffeoylquinic acid isomer	0.99
	40	Dicaffeoylquinic acid isomer	0.97
	55	Dicaffeoylquinic acid isomer	0.94
	3	Neochlorogenic acid	0.91
	33	Dicaffeoylquinic acid isomer	0.91
	5	Dihydroxybenzoic acid pentoside	0.88
	39	Apigenin-7-O-glucoside	0.88
	38	Dicaffeoylquinic acid isomer	0.88
	26	Mearnsetin-O-hexoside	0.86
	21	Feruloylquinic acid isomer	0.86
	23	Kaempherol-O-hexoside-deoxyhexoside	0.86
	43	Quercetin-O-caffeoylhexoside	0.86
Avu	38	Dicaffeoylquinic acid isomer	1.00
	37	Dicaffeoylquinic acid isomer	1.00
	34	Dicaffeoylquinic acid isomer	0.99
	33	Dicaffeoylquinic acid isomer	0.96

Only metabolites with pq(corr) > 0.85 are reported.

**Table 4:** Lists of metabolites that correlate with DPPH activity in *Artemisia* spp. samples.

Species	ID	DPPH—correlating metabolites	pq(corr)
Aab	15	Caffeoylquinic acid isomer	0.85
	9	Chlorogenic acid	0.85
Aal	38	Dicaffeoylquinic acid isomer	0.90
	37	Dicaffeoylquinic acid isomer	0.90
	34	Dicaffeoylquinic acid isomer	0.89
	9	Chlorogenic acid	0.86
Aan	34	Dicaffeoylquinic acid isomer	0.98
	40	Dicaffeoylquinic acid isomer	0.98
	21	Feruloylquinic acid isomer	0.97
	19	Feruloylquinic acid isomer	0.97
	50	Caffeoylferuloylquinic acid isomer	0.96
	37	Dicaffeoylquinic acid isomer	0.96
	9	Chlorogenic acid	0.96
	51	Caffeoylferuloylquinic acid isomer	0.96
	15	Caffeoylquinic acid isomer	0.96
	33	Dicaffeoylquinic acid isomer	0.94
	65	Tricaffeoylquinic acid isomer	0.94
	63	Diferuloylquinic acid isomer	0.93
	26	Mearnsetin-O-hexoside	0.92
	24	Quercetin-3-O-glucoside	0.90
	36	Isorhamnetin-3-O-Glucoside	0.88
	61	Dicaffeoylquinic acid isomer	0.87
	59	Caffeoylferuloylquinic acid isomer	0.86
Ave	34	Dicaffeoylquinic acid isomer	0.99
	37	Dicaffeoylquinic acid isomer	0.98
	40	Dicaffeoylquinic acid isomer	0.97
	33	Dicaffeoylquinic acid isomer	0.92
	55	Dicaffeoylquinic acid isomer	0.92
	31	Apigenin-O-hexoside-deoxyhexoside	0.90
	3	Neochlorogenic acid	0.89
	19	Feruloylquinic acid isomer	0.88
	26	Mearnsetin-O-hexoside	0.86
	38	Dicaffeoylquinic acid isomer	0.86
Avu	38	Dicaffeoylquinic acid isomer	0.86
	37	Dicaffeoylquinic acid isomer	0.86

Only metabolites with pq(corr) > 0.85 are reported.

Consistently to what observed in the previous paragraphs, the antioxidant activity of leaf extracts is in general higher than those of stem extracts (Fig. 7). This comparative analysis between the species, showed that the higher antioxidant activity of *A. verlotiorum*, described in the previous paragraph, may be mainly due to caffeic and ferulic acid derivatives and flavonoids; also, various unidentified metabolite showed high correlation with antioxidant activity (data not shown). In *A. vulgaris* the antioxidant activity correlated with caffeic acid derivatives, in A. *annua*, with coumaric, caffeic and ferulic acid derivatives. In *A. alba,* which showed the lowest linear correlation between metabolome composition and antioxidant activity (Fig. 7), also flavonoids were found to strongly correlate with antioxidant activity in FRAP assay, while the scavenging activity measured by DPPH was mostly correlated with dicaffeoylquinic acid isomers. Interestingly, coumarins accumulated at high levels in *A. alba* but did not strongly correlate with the antioxidant activity. Finally, in *A. absinthium* the antioxidant activity strongly correlated with caffeoylquinic acids, absinthin and flavonoids.

The hydroxycinnamates esterified with quinic acid, in particular some isomers of dicaffeoylquinic acid, were found to be metabolites with the strongest correlation with antioxidant activity in all species. This class of molecules has been extensively studied in the past years for their potential use in medicine. Caffeoylquinic acid derivatives are natural compounds isolated from a variety of traditional medicinal plants and possess a wide range of pharmacological properties, including antioxidant, hepatoprotective, antibacterial, antihistaminic and other biological effects^64^. Currently, in literature, caffeoyl and dicaffeoylquinic acids have been widely tested through in vitro and in vivo assays to evaluate their bioactive properties. Two caffeoylquinic acids extracted from *Aronia melanocarpa* berries, i.e. 3-caffeoylquinic acid and 4-caffeoylquinic acid, were identified as inhibitor of the dipeptidyl peptidase IV, an enzyme involved in the development of type 2 diabetes mellitus^65^. The protective effect of chlorogenic acid against neurotoxic effect of arsenic poisoning was demonstrated in mice model^66^. Potential benefits with therapeutic applications were reported also for dicaffeoylquinic acids. For example, Kim and collaborators demonstrated the neuroprotective effect of 3,5-dicaffeoylquinic acid and 3,4-dicaffeoylquinic acid from *Dipsacus asper* on hydrogen peroxide-induced cell death in SH-SY5Y human cells^67^. In another study it is reported that 1,5-dicaffeoylquinic acid (cynarin) downregulates the expression of inducible nitric oxide synthase, expressed under conditions of inflammation, sepsis, or oxidative stress, in human coronary smooth muscle cells^68^. In addition, the dicaffeoyl quinic acid cynarin affects the survival, growth, and stress response of normal, immortalized, and cancerous human cells^69^. Protective effects of cynarin against hepatoxicity effects of cyclophosphamide, an important anticancer drug which belongs to the class of alkylating agents, as well as its antihypertensive and vasodilator effects have been observed in in vivo studies^70,71^. *Artemisia* spp., which were able to accumulate various isomers of caffeoyl- and dicaffeoylquinic acid, could thus represent a valuable source of these potentially bioactive compounds. A further characterization to reveal the precise identity of the various isomers accumulated by the five species is thus required.

### Looking for a new artemisinin source

The sesquiterpene lactone artemisinin and its semi-synthetic derivatives are very important from a pharmaceutical perspective for their anti-malarial properties. Isolated from *A. annua* plants, artemisinin earned in short time the status of most potent antimalarial drug and recently new evidence of many other bioactivities (e.g. anticancer, anti-inflammatory and antiviral) have emerged^72^. For this reason, a great interest arose in the search for artemisinin-rich *A. annua* ecotypes and towards the manipulation of its biosynthetic pathway through different biotechnological tools^21^. Moreover, since antimalarial activity was reported for different *Artemisia* species^15^, many studies have been conducted to find alternative natural sources for artemisinin within the *Artemisia* genus. Despite artemisinin was demonstrated to occur in different amounts in *A. dubia*^73^, *A. scoparia*^74^, *A. cina*^75^, *A. vachanica* and *A. dracunculus*^76^, *A. verlotiorum* and *A. vulgaris*^77^, the major source of this metabolite still remains *A. annua*^78^.

**Figure 8:**
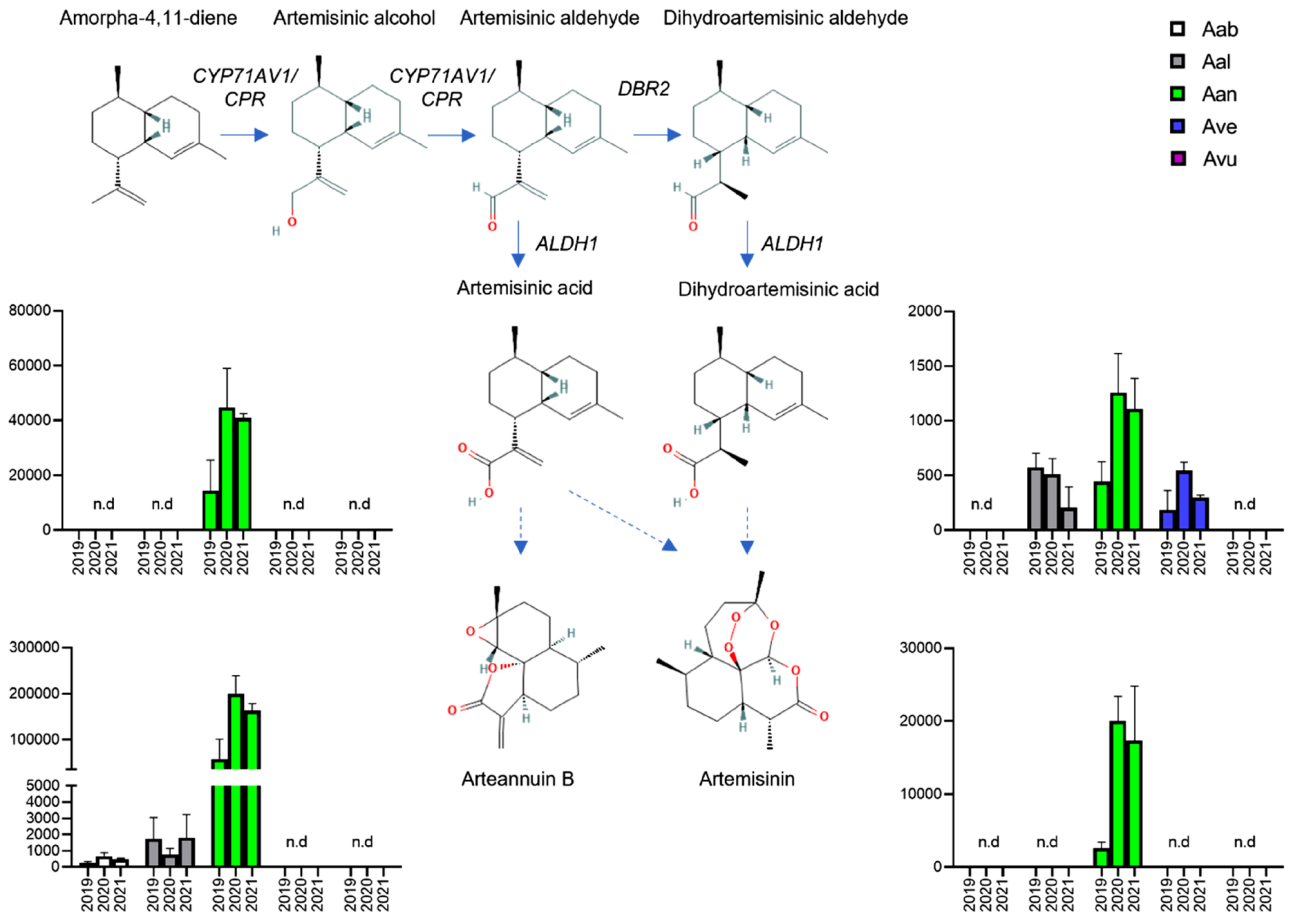
Relative comparison of the final sesquiterpenoid products from the artemisinin biosynthesis pathway in the five *Artemisia* species. Y axis: peak area arbitrary units. Bars represent SD (n = 3).

In this work we explored the capacity of the *Artemisia* plants collected in the province of Verona to produce the antimalaria lead drug artemisinin and related compounds from its biosynthesis pathway. We therefore performed an LC–MS analysis in positive ionization mode, which is more suitable for the ionization of sesquiterpenoid molecules, and we searched for the final products of the pathway (artemisinin and arteannuin B) and their immediate precursors (dihydroartemisinic acid and artemisinic acid, respectively). Their identification was made through the comparison of *m/z* values, fragmentation patterns and retention times with those of the respective reference standards (Table 5). The relative comparison of their levels within the leaves of the five *Artemisia* species is reported in Fig. 8.

**Table 5:** Metabolites from the artemisinin pathway with their MS features searched in LC–MS ESI^+^ analysis.

Metabolite	Formula	Rt (min)	Neutral mass (Da)	[M + H]^+^	[M + Na]^+^	MS/MS
Arteannuin B	C_15_H_20_O_3_	12.14	248.141	249.14	271.131	231.138 [M + H–H_2_O]; 185.134 [M + H–H_2_O–2CH_3_–O]
Artemisinin	C_15_H_22_O_5_	12.98	282.147	283.154	305.136	247.133 [M + H–2H_2_O]; 265.144 [M + H–H_2_O]
Artemisinic acid	C_15_H_22_O_2_	14.93	234.162	235.169		217.159 [M + H–H_2_O]; 199.149 [M + H–2H_2_O]
Dihydroartemisinic acid	C_15_H_24_O_2_	14.75	236.178	237.185		219.175 [M + H–H_2_O]

*A. annua* is the only species reporting detectable levels of artemisinin. Interestingly, the precursor of artemisinin, dihydroartemisinic acid, is present not only in *A. annua* but also in *A. alba* and *A. verlotiorum*. Arteannuin B, the final metabolite of a parallel pathway that originates from artemisinic aldehyde, was detected at high levels in *A. annua* and at considerably lower levels in *A. absinthium* and *A. alba* but was absent in *A. verlotiorum*. The precursor of both arteannuin B and artemisinin, artemisinic acid, is present in *A. annua*, as expected, but we did not detect it in *A. absinthium* nor *A. alba*, despite the fact that arteannuin B has been detected in both species. *A. vulgaris* does not produce any of the metabolites from the selected pathway.

According to the literature, the presence of artemisinin has been recently reported in *A. verlotiorum*^77^*, A. absinthium*^15^ and *A. vulgaris*^77^*.* While peak in artemisinin content in *A. annua* and *A. vulgaris* corresponds to the budding stage, in *A. absinthium* maximum accumulation is reached during the flowering stage^78–80^*.* This could explain the absence of artemisinin, or its eventual presence below detectable levels, in all the plants used in our work which were sampled during the vegetative growth. On the other hand, we reported for the first time the presence of artemisinin intermediates in *A. alba*, thus furtherly increasing the number of sesquiterpene compounds (e.g. germacrane and eudesmane) that were previously reported in this species^20^.

Although we did not detect traces of artemisinin, the presence of the precursor, dihydroartemisinic acid, and side product of the pathway, arteannuin B, indicates that genes of artemisinin biosynthetic pathway are all expressed in these plants, since the last step of artemisinin biosynthesis is a non-enzymatic photooxidative process^81^. The hypothesis that the artemisinin biosynthetic pathway may be an ancestral characteristic shared among many plants within genus *Artemisia*^15^ is supported by recent reports that confirmed the expression of structural genes in eight *Artemisia* species, including *A. absinthium* and *A. vulgaris*^78^. Their expression levels varied specifically depending on the organ collected and the developmental phase considered, with their expression ratio and turnover being crucial to address the flux of intermediates through the two branches of the pathway, leading in turn to artemisinin or arteannuin B accumulation. Nonetheless, the complexity of the physiological context linked to artemisinin accumulation recall the need to consider other factors to explain the large variability observed in the production of artemisinin-related metabolites^78^. For instance, the expression of transcription factors affecting structural genes and glandular trichome formation, which represent the site for artemisinin production and storage, have been recently investigated and considered as a target for metabolic engineering approaches to increase artemisinin levels^81^. In addition, the expression of other sesquiterpene synthases diverting the carbon resources of farnesyl-diphosphate into other competing pathways has to be considered.

Extending these transcriptomics analyses not only to *A. annua* but also to other species within the genus^82^ will provide useful molecular information to decipher, together with metabolomics data, the different biosynthetic capabilities of *Artemisia* spp. in artemisinin-related sesquiterpenes production and accumulation.

## Experimental section

### Plant material

The five selected *Artemisia* species were sampled independently throughout three growing seasons in June of the years 2019, 2020, 2021 from three hills and mountain areas in the province of Verona (Supplementary Table 3). Following identification, the plants were given the barcode number and a voucher specimen of each species is available at the “Museo di Storia Naturale” of Verona. All sampling procedures were conducted in accordance to the guidelines.

In each sampling site, plants were collected from three distinct spots (i.e. three different plant populations representing three biological replicates), far enough to avoid the sampling of plant populations deriving from the same genetic source. For each replicate, leaves and stems were collected from at least 5 individuals and pooled together according to the organ. The samples were immediately frozen in dry ice and then stored at − 80 °C. The frozen plant material was homogenized in liquid nitrogen using an IKA A11 basic mill (IKA, Germany).

### Chemicals and reagents

Reference standard of artemisinin was purchased from Sigma-Aldrich (St. Louis, USA). Reference standards of arteannuin B and artemisinic acid were purchased from Biosynth® Carbosynth (Bratislava, Slovakia). Reference standard of dihydroartemisinic acid was purchased from Toronto Reasearch Chemical (Toronto, Canada). Methanol, acetonitrile and water (all LC–MS grade) were purchased from Honeywell (Charlotte, USA). Formic acid (LC–MS grade) was purchased from Biosolve Chimie (Dieuze, France). Trolox and DPPH were purchased, respectively, from Sigma-Aldrich and Thermo Fisher Scientific.

### Metabolites extraction

100 mg of frozen powder were extracted in 1.5 ml methanol, mixed vigorously for 30 s, sonicated at 40 kHz in an ultrasonic bath Sonica® Ultrasonic Cleaner (SOLTEC, Milan, Italy) at 4 °C for 15 min and centrifuged (16,000*g*, 15 min, 4 °C). The supernatants were opportunely diluted in 100% methanol. Just before the analysis, the samples were diluted 1:2 (V:V) with ultrapure water (Honeywell, USA). The final mixtures were passed through Minisart RC4 filters (0.2 μm pores) (Sartorious, Göttingen, Germany) and 1 μl was injected into the UPLC device.

### UPLC–ESI–MS analysis

An Acquity I Class UPLC system (Waters, Milford, USA) with a BEH C18 column (Waters), coupled online with a PDA (photo-diode array) and to a Xevo G2-XS qTOF mass spectrometer (Waters), equipped with an electrospray ionization (ESI) source were used. The extracts were injected through a cooled autosampler (8 °C) and a flow rate of 0.350 ml/min was used. The mobile phases were 0.1% formic acid in water (solvent A) and acetonitrile (solvent B), and the elution gradient was as follows: 0–1 min, 1% B; 1–10 min, 1–40% B ; 10–13.50 min, 40–70% B; 13.50–15.00 min, 70–90% B; 15.00–16.50 min, 90–100% B; 16.50–20 min 100% B; 20–20.1, 100–1% B; 20.1–25 min, 1% B (initial conditions).

The sample analysis sequence was randomized. A quality control (QC) prepared by mixing equal part of all the extracts was analyzed along the whole experiment every ten sample analysis. The ion source parameters were the following: capillary voltage 0.8 kV, sampling cone voltage 40 V, source offset voltage 80 V, source temperature 120 °C, desolvation temperature 500 °C, cone gas flow rate 50 l/h and desolvation gas flow rate 1000 l/h. Nitrogen gas was used for the nebulizer and in desolvation whereas argon was used to generate collision-induced dissociation. MS data were acquired in continuum in both negative and positive ionization mode within the range 50–2000 *m/z* using a fixed collision energy of 35 V. Data were acquired through the Mass Lynx v4.2 software (Waters).

### Processing of LC–MS data and metabolites identification

The chromatograms were manually inspected through Mass Lynx software. Metabolites were identified by relying on *m/z* value of the monoisotopic molecular ion, retention time and MS/MS fragmentation pattern by comparison with an in-house library of authentic standard. When no standard compounds were available, the identification was tentatively assigned comparing *m/z*, isotopic ratio, fragmentation pattern and UV/vis absorbance spectra with those reported in scientific literature and public databases (Chemspider, Human Metabolome Database, Lotus Natural Products, MassBank, MoNA, Pubchem, etc.). In particular, for the characterization of caffeoyl ester derivatives and various glycosides the following neutral losses (Da) were considered: 132.042 (pentose), 146.058 (deoxyhexose), 162.032 (caffeic acid moiety), 162.053 (hexose),

The chromatograms acquired in negative ionization mode were processed with Progenesis QI software (Waters) to obtain the Feature Quantification Matrix (FQM; Supplementary File 1).

### Antioxidant assays

The same methanolic extracts used for UPLC-ESI–MS analysis were used for determination of antioxidant activity in vitro by FRAP and DPPH assays in transparent 96-well microplates.

A FRAP solution was prepared mixing in a ratio of 10:1:1 (V:V:V) the following reagents: FRAP buffer (3.1 g/l sodium acetate trihydrate, 16 ml/l acetic acid pH 3.6), 10 mM TPTZ (2,4,6-tri(2-pyridyl)-1,2,5-triazine) in HCl 40 Mm, FeCl_3 _* 6H_2_O 20 Mm , The test was carried out mixing 200 µl of the FRAP solution to 20 µl of the sample, or solutions of Trolox at different concentrations or methanol (blank). Methanolic extracts of samples were diluted 1:20 for leaves and in a range from 1:3 to 1:10 for stems. Each sample was tested in three technical replicates. The microplate was incubated at 37 °C in the dark for 15 min and then kept cooling at room temperature for 4 min. The absorbance was measured at 593 nm using the Infinite 200 PRO plate reader (Tecan, Männedorf, Switzerland).

1 mM DPPH stock solution was freshly prepared in methanol at least 2 h before the assay. 100 µM of working solution was prepared diluting 1:10 (V:V) in 70% methanol the DPPH solution. 200 µl of the DPPH solution were added to 20 µl of the sample, i.e. diluted plant extracts or solutions of Trolox at different concentrations or methanol (blank). Methanolic extracts were diluted in a range from 1:10 to 1:20 and from 1:3 to 1:10 for leaves and stems, respectively. Each sample was tested in three technical replicates. The microplate was incubated at 25 °C in the dark for 30 min and then the absorbance was measured at 517 nm using the Infinite 200 PRO plate reader (Tecan).

The compound Trolox, a water-soluble Vitamin E analogue, was used as reference antioxidant in order to express the antioxidant power of the plant extracts, expressed as Trolox Equivalent Antioxidant Capacity (TEAC), whose unit is mmol of Trolox Equivalent for Kg (mmol/kg). 20 µl of Trolox solutions with concentrations spanning from 500 to 5 µM was added to 200 µl of FRAP or DPPH solution to generate a Trolox calibration curve in each assay.

### Statistical analysis

The FQM and antioxidant (TEAC values) data were analyzed with SIMCA-P software (Umetrics, Sweden) for multivariate statistical analysis in order to look for relationships among the in-vitro antioxidant activity of the plant extracts and their metabolite composition. The *m/z* features (i.e. the metabolites) of the dataset were assigned as X variables (Pareto scaling) and the antioxidant activity as Y variables (UV scaling). Orthogonal Partial Least Square (OPLS) analysis was used. Metabolites putatively responsible for the antioxidant activity were identified by inspection of the column loading plot; only metabolites showing a pq(corr) value > 0.8 (arbitrary threshold) were considered correlated with antioxidant activity. All statistical calculations were performed using the GraphPad Prism version 8.0 software (GraphPad Software, San Diego, California USA). The means values ± SD (n = 3) are reported in the figures. Statistical analyses were conducted using One or Two-way Anova followed by Tukey’s Test.

### Supplementary Information

Supplementary Information 1.

Supplementary Information 2.

Supplementary Information 3.

Supplementary Information 4.

Supplementary Information 5.

## Data Availability

All data generated or analysed during this study are included in this published article and its supplementary information files.
