# Author Correction: Bioprospecting of *Artemisia* genus: from artemisinin to other potentially bioactive compounds

**DOI:** 10.1038/s41598-024-63761-x

**Published:** 2024-06-06

**Authors:** Stefano Negri, Fabio Pietrolucci, Sebastiano Andreatta, Ruth Chinyere Njoku, Carolina Antunes Silva Nogueira Ramos, Massimo Crimi, Mauro Commisso, Flavia Guzzo, Linda Avesani

**Affiliations:** 1 Department of Biotechnology, University of Verona, 15, Strada Le Grazie, 37134 Verona, Italy; 2 National Biodiversity Future Center (NBFC), 90133 Palermo, Italy; 3 Museo di Storia Naturale, Comune di Verona, 37126 Verona, Italy

Correction to: *Scientific Reports* 10.1038/s41598-024-55128-z, published online 27 February 2024

The original version of this Article contained errors in the Author Contributions section.

“S.N., F.P., S.A., C.R., L.A. performed the sampling. S.N., R.N. performed the bioactivity tests, M.C. and Ma. Cr., revised the manuscript, F.G. and L.A. designed the work. S.N., L.A. and F.G. wrote the first draft of the manuscript.”

now reads:

“S.N., F.P., S.A., C.R., L.A. performed the sampling. F.P. and S.N. performed metabolomic analysis; S.N., R.N. and F.P. performed the bioactivity tests, M.C. and Ma. Cr., revised the manuscript, F.G. and L.A. designed the work. S.N., L.A., F.P. and F.G. wrote the manuscript.”

The original Article has been corrected.

