# Supplementary material for: Bioprospecting of Artemisia genus: from artemisinin to other potentially bioactive compounds
Source: Sci Rep. 2024 Feb 27;14:4791. doi: 10.1038/s41598-024-55128-z (PMC10899597; doi:10.1038/s41598-024-55128-z)
Supplement: Supplementary file 2 — Supplementary Information 2. [file 41598_2024_55128_MOESM2_ESM.docx]

**Supplementary Table 2**. Antioxidant activity of *Artemisia* spp. methanolic extracts assayed by various authors (the original values reported in the articles have been converted to mmolTE/Kg for comparison with our data; na, not available).

|  | FRAP  (mmol TE/Kg dry wt) | DPPH  (mmol TE/Kg dry wt) | Reference |
| --- | --- | --- | --- |
| *A. absinthium* | 269.5 ± 14.2 | 341.0 ± 4.8 | [15] |
| *A. alba* | na | 116.3 – 427.5 | [45] |
| *A. annua* | 410.1 ± 8.6 | 234.4 ± 5.8 | [15] |
| *A. verlotiorum* | 973.3 ± 2.7 | 769.4 ± 2.3 | [46] |
| *A. vulgaris* | 557.6 ± 12.7 | 793.1 ± 20.0 | [15] |
