# Supplementary material for: Bioprospecting of Artemisia genus: from artemisinin to other potentially bioactive compounds
Source: Sci Rep. 2024 Feb 27;14:4791. doi: 10.1038/s41598-024-55128-z (PMC10899597; doi:10.1038/s41598-024-55128-z)
Supplement: Supplementary file 3 — Supplementary Information 3. [file 41598_2024_55128_MOESM3_ESM.docx]

**Supplementary Table 1**. TEAC mean values (mmol TE/ Kg FW) of FRAP and DPPH, for each species leaves and stem are reported with the three different years of sampling. Values are expressed as mean ± standard deviations

|  |  |  | **FRAP (mmol TE/Kg fr. wt)** | | | | **DPPH (mmol TE/Kg fr. wt)** | | | |
| --- | --- | --- | --- | --- | --- | --- | --- | --- | --- | --- |
|  |  |  |  |  |  | **average of 3 years** |  |  |  | **average of 3 years** |
| **Aab** | leaves | 2019 | 22.3 | ± | 8.2 |  | 21.7 | ± | 10.0 |  |
|  |  | 2020 | 36.5 | ± | 4.1 | 30.0 ± 7.2 | 26.9 | ± | 2.9 | 24.8 ± 2.7 |
|  |  | 2021 | 31.1 | ± | 4.1 |  | 25.8 | ± | 0.8 |  |
|  | stems | 2019 | 14.6 | ± | 11.3 |  | 17.2 | ± | 16.9 |  |
|  |  | 2020 | 17.1 | ± | 6.7 | 16.8 ± 2.1 | 12.5 | ± | 3.3 | 16.0 ± 3.0 |
|  |  | 2021 | 18.7 | ± | 0.3 |  | 18.2 | ± | 1.0 |  |
| **Aal** | leaves | 2019 | 80.0 | ± | 19.3 |  | 64.1 | ± | 14.4 |  |
|  |  | 2020 | 94.7 | ± | 19.9 | 86.5 ± 7.5 | 56.1 | ± | 14.1 | 61.9 ± 5.0 |
|  |  | 2021 | 84.7 | ± | 19.9 |  | 65.4 | ± | 14.1 |  |
|  | stems | 2019 | 48.8 | ± | 9.8 |  | 36.8 | ± | 11.4 |  |
|  |  | 2020 | 62.6 | ± | 12.1 | 59.7 ± 9.7 | 58.6 | ± | 16.8 | 51.4 ± 12.6 |
|  |  | 2021 | 67.6 | ± | 16.3 |  | 58.8 | ± | 9.6 |  |
| **Aan** | leaves | 2019 | 30.7 | ± | 8.9 |  | 21.9 | ± | 3.8 |  |
|  |  | 2020 | 97.5 | ± | 2.0 | 73.0 ± 36.8 | 71.5 | ± | 8.4 | 51.8 ± 26.3 |
|  |  | 2021 | 90.7 | ± | 7.4 |  | 61.9 | ± | 7.0 |  |
|  | stems | 2019 | 17.8 | ± | 3.8 |  | 14.2 | ± | 3.9 |  |
|  |  | 2020 | 91.6 | ± | 16.7 | 49.8 ± 37.9 | 51.4 | ± | 7.8 | 32.6 ± 18.6 |
|  |  | 2021 | 39.9 | ± | 7.8 |  | 32.3 | ± | 5.3 |  |
| **Ave** | leaves | 2019 | 94.1 | ± | 16.2 |  | 69.6 | ± | 10.1 |  |
|  |  | 2020 | 94.2 | ± | 11.2 | 121.2 ± 46.9 | 66.7 | ± | 3.6 | 88.7 ± 35.6 |
|  |  | 2021 | 175.3 | ± | 16.8 |  | 129.7 | ± | 1.2 |  |
|  | stems | 2019 | 49.7 | ± | 3.9 |  | 39.8 | ± | 8.3 |  |
|  |  | 2020 | 77.1 | ± | 12.1 | 72.5 ± 20.9 | 50.6 | ± | 9.5 | 54.9 ± 17.6 |
|  |  | 2021 | 90.7 | ± | 15.6 |  | 74.3 | ± | 2.0 |  |
| **Avu** | leaves | 2019 | 62.6 | ± | 14.1 |  | 33.7 | ± | 5.9 |  |
|  |  | 2020 | 86.2 | ± | 21.1 | 80.5 ± 15.8 | 56.5 | ± | 17.5 | 52.0 ± 16.5 |
|  |  | 2021 | 92.6 | ± | 10.2 |  | 65.7 | ± | 5.6 |  |
|  | stems | 2019 | 23.7 | ± | 10.0 |  | 17.1 | ± | 4.8 |  |
|  |  | 2020 | 67.0 | ± | 14.6 | 43.7 ± 21.8 | 43.2 | ± | 11.5 | 31.6 ± 13.3 |
|  |  | 2021 | 40.5 | ± | 4.5 |  | 34.4 | ± | 3.6 |  |
