# Supplementary material for: Bioprospecting of Artemisia genus: from artemisinin to other potentially bioactive compounds
Source: Sci Rep. 2024 Feb 27;14:4791. doi: 10.1038/s41598-024-55128-z (PMC10899597; doi:10.1038/s41598-024-55128-z)
Supplement: Supplementary file 5 — Supplementary Information 5. [file 41598_2024_55128_MOESM5_ESM.docx]

**Supplementary Table 3.** Resume of plant species and organs sampled with geolocation coordinates and herbarium deposit number.

| Species | Location of sampling | Herbarium voucher number |
| --- | --- | --- |
| *A. absinthium* | 45°38'45.1"N 11°05'22.5"E  Conca dei Parpari (Roverè Veronese) | VER n.SA625 |
| *A. alba* | 45°35'23.2"N 11°05'03.8"E  Vazzi (Roverè Veronese) | VER n.SA627 |
| *A. annua* | 45°27'34.8"N 11°03'00.8"E  Castle of Montorio (Verona) | VER n.SA623 |
| *A. verlotiorum* | 45°27'36.0"N 11°02'59.5"E  Castle of Montorio (Verona) | VER n.SA624 |
| *A. vulgaris* | 45°35'23.4"N 11°05'01.4"E  Vazzi (Roverè Veronese) | VER n.SA626 |
